# Predicting Subtype Selectivity for Adenosine Receptor Ligands with Three-Dimensional Biologically Relevant Spectrum (BRS-3D)

**DOI:** 10.1038/srep36595

**Published:** 2016-11-04

**Authors:** Song-Bing He, Ben Hu, Zheng-Kun Kuang, Dong Wang, De-Xin Kong

**Affiliations:** 1 State Key Laboratory of Agricultural Microbiology, Huazhong Agricultural University, Wuhan 430070, China; 2 College of Life Science and Technology, Huazhong Agricultural University, Wuhan 430070, China; 3 Agricultural Bioinformatics Key Laboratory of Hubei Province, College of Informatics, Huazhong Agricultural University, Wuhan 430070, China

## Abstract

Adenosine receptors (ARs) are potential therapeutic targets for Parkinson’s disease, diabetes, pain, stroke and cancers. Prediction of subtype selectivity is therefore important from both therapeutic and mechanistic perspectives. In this paper, we introduced a shape similarity profile as molecular descriptor, namely three-dimensional biologically relevant spectrum (BRS-3D), for AR selectivity prediction. Pairwise regression and discrimination models were built with the support vector machine methods. The average determination coefficient (*r*^2^) of the regression models was 0.664 (for test sets). The 2B-3 (A_2B_ *vs* A_3_) model performed best with *q*^2^ = 0.769 for training sets (10-fold cross-validation), and *r*^2^ = 0.766, *RMSE* = 0.828 for test sets. The models’ robustness and stability were validated with 100 times resampling and 500 times Y-randomization. We compared the performance of BRS-3D with 3D descriptors calculated by MOE. BRS-3D performed as good as, or better than, MOE 3D descriptors. The performances of the discrimination models were also encouraging, with average accuracy (ACC) 0.912 and *MCC* 0.792 (test set). The 2A-3 (A_2A_ *vs* A_3_) selectivity discrimination model (*ACC* = 0.882 and *MCC* = 0.715 for test set) outperformed an earlier reported one (*ACC* = 0.784). These results demonstrated that, through multiple conformation encoding, BRS-3D can be used as an effective molecular descriptor for AR subtype selectivity prediction.

Adenosine receptors (ARs) belong to the G protein-coupled receptors (GPCRs) superfamily. ARs include four subtypes, referred to as A_1_, A_2A_, A_2B_, and A_3_. These subtypes have been identified in different tissues from several mammalian species, including human^1^,^2^. ARs mediate the physiological actions of adenosine and therefore are potential therapeutic targets for Parkinson’s disease, diabetes, pain, stroke and different kinds of cancer^3^. A_1_ selective antagonists have anxiolytic effect and were reported as promising candidates for the treatment of cognitive disorders, such as dementia^4^. Selective antagonism of A_1_ was also proposed as mechanism for some diuretic agents. The agents were effective in the treatment of congestive heart failure and edema^5^. A_2A_ antagonists have neuro-protective activity during the ischemic process and reduce the neuronal damage of Parkinson’s or Huntington’s diseases^6^,^7^,^8^. A potential therapeutic activity of asthma disease was discovered for A_2B_ selective antagonists or mixed antagonists to A_2B_ and A_3_^6^,^9^. A_2B_ antagonists are also studied as hypoglycemic agents in diabetes, while A_3_ antagonists have a potential application in tumor growth inhibition and in the treatment of glaucoma^6^.

The four AR subtypes have different tissue distribution and pharmacological profile. A_1_ and A_2A_ possess high affinity to adenosine, while A_2B_ and A_3_ show relatively lower affinity^10^. A_1_ and A_3_ are coupled to G_i/o_ proteins to inhibit adenylate cyclase and consequently decrease the production of cyclic AMP (cAMP), while A_2A_ and A_2B_ stimulate the production of cAMP by coupling to G_s/o_ proteins^6^. These two subtype pairs share higher sequence identity. The sequence identity of human A_1_ and A_3_ is 49%, while the identity of A_2A_ and A_2B_ is 59%^11^.

Adenosine signaling is widespread throughout the body and the receptors exerts a broad spectrum of physiological and pathophysiological functions through adenosine binding^6^. Therefore, AR subtypes selectivity is highly desired in developing therapeutic agents with minimal side effects^12^. However, the sequences and binding pocket structures of the AR subtypes are highly similar to each other. These pose a great challenge to subtype selective ARs ligands design.

Approaches of rational drug design can be adopted to reduce the arbitrariness in selective ligands screening. In 2011, Katritch *et al*. reported their structure-based study on subtype-selectivity of ARs antagonists^12^. The structures of A_1_, A_2B_, A_3_ were built by comparative modeling, taking the crystal structure of A_2A_ as a template, which was the only known structure of AR subtypes in PDB^13^. However, application of structure-based methods is limited by the accuracy of homology modeled structures, docking efficiency and scoring function precision.

Ligand-based methods, especially quantitative structure-activity relationships (QSARs), can be adopted in the absence of target structural information. In fact, QSAR played an indispensable role in GPCR subtype selective ligand design^14^,^15^, e.g., ARs^16^, dopamine receptors^17^, serotonin receptors 5HT1E/5HT1F^18^ and cannabinoid receptor CB1/CB2^19^,^20^. For AR ligands, Michelan *et al*. introduced a multi-label classification approach, the so-called cross-training with SVM (ct-SVM), to derive compound potency profiles against human AR subtypes and to predict the selectivity^16^. They further applied SVM classification and regression in combination in predicting the selectivity profiles of adenosine A_2A_ and A_3_ antagonists and their binding affinities^21^. After leave-one-out (LOO), 10-fold and 5-fold cross-validation process, they achieved an over-all prediction accuracy 78.4% for the test set, confirmed the statistical reliability of this model^21^. Two regression models for A_2A_ and A_3_ antagonistic activity prediction yielded correlation coefficients 0.78 and 0.85, respectively, after LOO cross-validation^21^,^22^.

Recently, we developed a multiple dimensional molecular descriptor, namely three-dimensional biologically relevant spectrum (BRS-3D)^23^. BRS-3D was calculated by superimposing the molecule under investigation against 300 template molecules that were diversely extracted from the crystalized ligands in PDB database. Then, information about the molecules’ multiple conformations can be encoded into the 300 dimensional molecular descriptor. We believe that BRS-3D can be well applied to GPCR subtype selectivity prediction. In this paper, predictive regression and discrimination AR subtype selectivity models were successfully built with machine learning method, support vector machine (SVM).

## Materials and Methods

### Data set preparation

All structural and activity data were retrieved from the ChEMBL database (release 20)^24^. The dataset was filtered according to the following criteria: the target is derived from *homo sapiens*; the target is a single protein and the assay for the target is a binding assay^25^. Minus logarithm binding affinities (p*K*_i_ value) were used to measure how well a compound binds to ARs. Only compounds with explicitly defined potency were retained. Entries with activity annotations such as “>”, “<” or “~” were discarded. For these compounds with more than one reported activities, average p*K*_i_ values were calculated and used. It should be noted that the ChEMBL dataset were carried out by different research groups with different experimental conditions. The lack of homogeneity and clear ontology of the activity data made ARs selectivity prediction a challenge. However, we believed that only through such a big-data study, could we find the real structure-selectivity relationships of the diverse ARs ligands.

**Figure 1:**
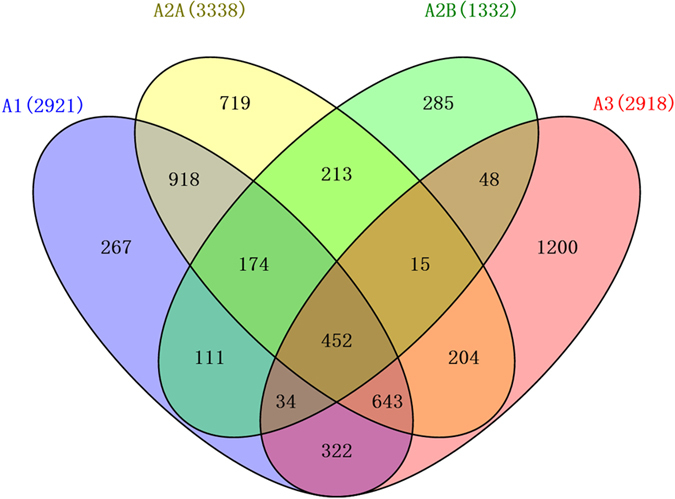
Venn diagram of the available ARs activity data from ChEMBL. Compounds were filtered for homo species single proteins with p*K*_i_ data. The compounds that coexisted in two subtypes were used in building the pairwise selectivity regression models. Among them, selective compounds (with |*SR*| > 1) were used for the pairwise discrimination models.

The structures were standardized using an in-house Pipeline Pilot protocol (version 8.5)^26^. Hydrogen was added to fulfill the valences of heavy atoms and neutralize the molecular charge. Molecules with less than 8 or more than 80 heavy atoms were eliminated. After the prescreening process, 1332 (A_2B_) to 3338 (A_2A_) molecules were retained in the data sets (Fig. 1). The amounts of active compounds of different subtypes were in the same order of magnitude. Sufficient active molecules and balanced distribution of them in the four AR subtypes are conducive to the theoretical modeling. At last, the structures were converted into three dimensional conformations with CONCORD module and minimized with Tripos force field and default parameters in SYBYL-X 2.0^27^. The distributions of p*K*_i_ and some physicochemical properties of the compounds were shown in Supplementary Figure S1. The structures, ChEMBL ID, pKi affinities to ARs, selectivity ratios and BRS-3D features were provided in a zipped sdf file in the Supplementary Information.

The four AR subtypes formed six pairwise data sets, namely 1-2A (A_1_ *vs* A_2A_, similarly hereinafter), 1-2B, 1-3, 2A-2B, 2A-3 and 2B-3. These data sets were demonstrated with the intersection of two colors in Fig. 1. The selectivity ratio (*SR*) was defined as *SR*_T1-T2_ = p*K*_iT1_-p*K*_iT2_, for AR subtypes T1 and T2. Through this way, a positive *SR* value indicates that the compounds have a higher binding potency to T1 than T2, and vice versa. For subtype selectivity regression model, we used *SR* directly as the dependent variable. For subtype selectivity discrimination model, compounds with *SR* greater than 1 or less than −1 were defined as selective agents^28^,^29^. A *SR* equal to 1 indicates that the compound can bind to T1 with a potency 10-fold higher than to T2.

For all the data sets, molecules were randomly grouped into training sets and test sets at a ratio of 4:1. The training sets (80%) were used to develop the prediction models, while the test sets (20%) were used to assess the performance of the models.

### Molecular descriptor, BRS-3D

**Figure 2:**
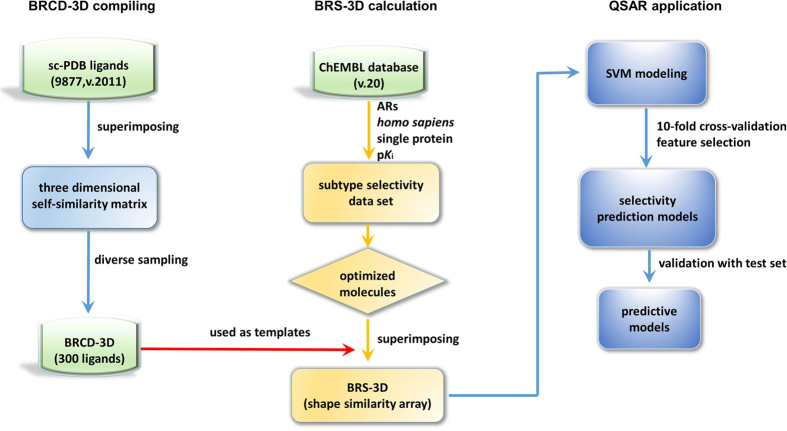
Flowchart of selectivity prediction workflow based on BRS-3D. There are three steps for a BRS-3D modeling. (1) BRCD-3D compiling. Based on the self-similarity matrix between all the ligand pairs in sc-PDB, 300 ligands (BRCD-3D) were diversely selected with cluster analysis. The sc-PDB database was employed here as a representative collection of known bioactive conformations. (2) BRS-3D calculation. BRS-3D is a shape similarity profile calculated with molecular superimposition. The molecules under scrutiny were superimposed onto the 300 templates (BRCD-3D) and resulted into a 300 dimensional array. The shape similarity array was defined as BRS-3D. (3) QSAR application. Using BRS-3D as molecular descriptor, QSAR models can be developed with various statistical methods.

Molecular descriptors are characterization of the molecules’ structural and physicochemical properties. We used a novel multi-dimensional molecular descriptor, BRS-3D, which is a shape similarity profile calculated with molecular superimposition. It was named after our previous two-dimensional approach^30^. The procedure of using BRS-3D in QSAR study was illustrated in Fig. 2.

First, a database was constructed with 300 ligands which were diversely selected from sc-PDB (version 2011, http://bioinfo-pharma.u-strasbg.fr/scPDB/). This database was named 3D bio-relevance representative compounds database (BRCD-3D). We used sc-PDB because it is a focused “drug-like” subset of the original PDB^31^. Some of the sc-PDB ligands existed in more than one complexes. It is unnecessary and computationally wasteful to use all the ligands as templates. Diverse sampling can be used to reduce the redundancy. Comparison showed that BRCD-3D with 300 ligands performed similarity to the results with 500 ligands while it saved lots of calculation expenditure (unpublished data). The 300 diverse templates were extracted by cluster analysis based on the self-shape-similarity matrix of all 9878 ligands in sc-PDB. The self-shape-similarity were calculated with Surflex-Sim rigid superimposing. Then, the molecule under scrutiny was superimposed onto the 300 templates and resulted into a 300-dimensional similarity array (BRS-3D). Since the 300 ligands were diversely selected, they can act as the landmark in the biologically active conformation space. BRS-3D can be used as a “GPS” system in such a space. Elements in BRS-3D reflect the shape and electrostatic properties of the objective molecule, and then can be used as a descriptor in QSAR or virtual screening.

BRS-3D calculation was performed by an in-house shell script. We used Surflex-Sim, a module of Surflex suite in SYBYL-X 2.0, for molecular superimposition and shape similarity calculation. Surflex-Sim overlay two molecules and quantify the 3D similarity with the morphological similarity algorithm. The similarity scores ranged from 0 to 1. 10 superimposed conformations and similarity scores between the objective molecule and a template would be obtained. Only the highest score was selected as an element of BRS-3D. The similarity score takes into account both the match of surface shape and charge characteristics of the objective molecules^32^,^33^.

### 3D molecular descriptors in MOE

We compared the performances of BRS-3D and three dimensional (3D) molecular descriptors calculated with MOE (version 2014). The MOE 3D descriptors comprised 91 surface area, volume and shape related properties. Detailed list of MOE 3D descriptors can be found in Supplementary Table S1.

### Model development

The widely used machine learning method SVM was employed to develop the prediction models. SVM was originally proposed by Vapnik *et al*.^34^. This method can be used to solve both classification and regression problems. We used the SVM embedded in “e1071” package from R, invoked through R statistics module in Pipeline Pilot 8.5^35^. According to reported literatures, SVM are among the best-performing approaches for chemical and biological property prediction and the computational identification of active compounds^35^. SVM projects the data into a higher dimensional feature space where linear separation is frequently possible, facilitating object classification, ranking and regression-based property value prediction. Radial basis function (RBF) kernel was used to obtain a complicated nonlinear separating hyperplane. A key feature of SVM is that it attempts to minimize the error on training data and reduce the computational complexity of models to avoid over-fitting by using the structural risk minimization. Furthermore, projection of BRS-3D features in a multi-dimensional space with kernel functions avoided heavy explicit calculation.

A 10-fold cross-validation on the training set was performed to determine the optimal parameter settings (gamma γ for the RBF kernel and “C” value of the constant for the slacks variant) with grid searching. Other parameters were set to their default values.

### Feature selection

Presence of irrelevant or redundant features could cause over-fitting and poor generalization capacity of the developed models. As an important step, feature selection can prune the irrelevant and redundant information and improve the performance of learning algorithms^36^. Identifying the most relevant features can effectively remove the irrelevant data, reduce the issue dimensionality, increase learning performance and improve the result comprehensibility. Random forest (RF) was used for feature selection. RF was a popular and efficient algorithm, based on model aggregation ideas, regardless of classification or regression problems^37^. RF was implemented by the component “Learn R Forest Model” in Pipeline Pilot 8.5, invoking the R package “RandomForest”. The principle of RF is to combine many binary decision trees, which were built with bootstrap on the training sample and random selection of explanatory variables at each node^38^. After ranking variables by the importance, only those top-ranking features were retained for model construction. We compared the performance of 8 feature subsets with the top 3 (1%), 15 (5%), 30 (10%), 60 (20%), 120 (40%), 180 (60%), 240 (80%) and all the 300 (100%) features.

The prediction accuracies of different feature subsets were compared according to the proportion of correctly classified samples in discriminant models, or the correlation between the predicted and actual selectivity values in regression models. We also studied the influence of feature selection on models’ performance with the test set (20% random sample from the original data set). Of course, the compounds in test set were only used for the purpose of model evaluation.

### Model performance assessments

For the regression models, we used cross-validation determination coefficient (*q*^2^, Formula 1, for training set), the root-mean-square error (*RMSE*, Formula 2) and determination coefficient (*r*^2^, Formula 3, for test set) as a measure of model fitting and predictive power^39^. *q*^2^ takes values in a standardized range, thus allowing easily comparison of different QSAR models, fitting performance and model predictive abilities^40^. *RMSE*, an equivalent measure of dispersion, is a helpful indicator of a model’s usefulness^41^. *r*^2^ is defined as the square of the correlation coefficient between the observed and predicted values in a regression. The formulae for the calculation of these parameters were as follows.


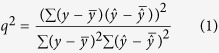



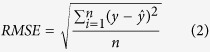



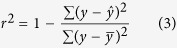


Where *n* stands for the total number of compounds, *y* is the observed response variables, 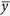 is the mean of *y*, and 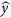 is the predicted value.

The quality of all discrimination models was evaluated by considering the following statistical indicators: sensitivity (*SE*), specificity (*SP*), overall prediction accuracy (*ACC*) and Matthews correlation coefficient (*MCC*) (Formulae 4–7). Furthermore, we used the receiver-operating characteristic (ROC) and the area under the ROC (*AUC*) as advocated by Nicholls^42^. *AUC*_*cv*_ was also used in cross-validation (CV) as the indicator in the grid parameter searching.


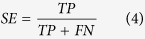



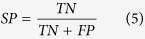



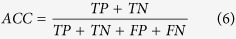



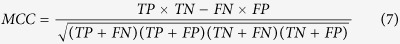


Here, *TP*, *FP*, *TN* and *FN* represent true positives, false positives, true negatives, and false negatives, respectively.

### Y-randomization test

Y-randomization test was carried out to exclude the possibility of chance correlation^43^. The *SR* values (response variable) were randomly shuffled to change their true order. Thus, although the *SR* values (and the statistical distribution) stayed the same, their position against the appropriate compound and its descriptors were now altered. This process was repeated for 500 times.

### Applicability domain evaluation

Applicability domain (AD) evaluation is one of the most important part in QSAR modeling^44^. In the study, the Williams plot based on standardized residuals and leverage values was used to define the AD of the AR subtype selectivity prediction models. Williams plot provides leverage values plotted against the prediction errors. Both the structural outside compounds (h > h*) and response outliers (standardized residuals >3 or < −3) can be detected. The leverage value (*h*) measures the distance from the centroid of the modeled space and could be calculated for a given data set **X** by obtaining the Hat matrix (**H**) by Formula 8^45^:


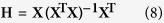


where **X** is the selected descriptors matrix; **X**^**T**^ is the transpose matrix of **X**; and **(X**^**T**^**X)**^**−1**^ is the inverse of matrix **(X**^**T**^**X)**. The leverages of the compounds in the data set are the diagonal elements of the **H** matrix. The warning leverage (h*) is generally calculated as h* = 3*p/n*, where *p* is the number of variables plus one and *n* is the number of samples in training set. If a compound in the test set has a leverage value higher than h*, it is considered outside the AD and its prediction result may be unreliable.

## Results

### Pairwise subtype selectivity regression models

**Figure 3:**
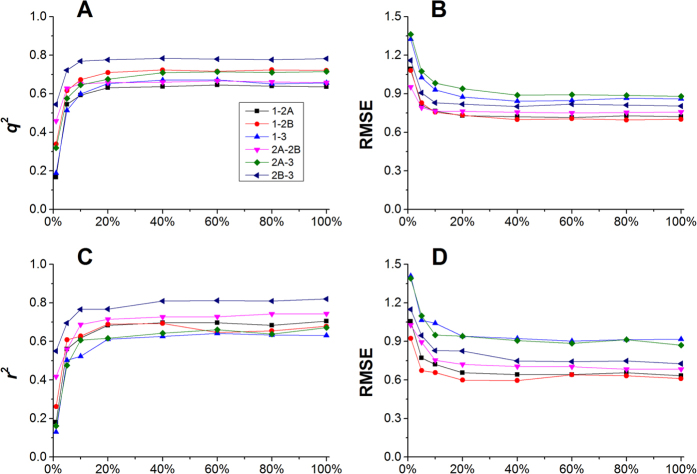
Feature selection results of the six pairwise regression models. (**A**) *q*^2^ of the training sets. (**B**) *RMSE* of the training sets. (**C**) *r*^2^ of the test sets. (**D**) *RMSE* of the test sets. Eight different feature subsets were explored. The training sets were calculated based on 10-fold cross-validation. The test sets were used only for model evaluation.

Six pairwise regression models were successfully constructed. Feature selection (Fig. 3) showed that the performances of the models rose greatly when the employed features increased from 1% to 20%. The results indicated that around 60 features were related to subtype selectivity. When more than 20% features were included, the models’ statistical parameters became stable.

According to Golbraikh’s suggestion, regression models with cross-validated *r*^2^ (*q*^2^) value for the training set greater than 0.5 and linear fit predictive *r*^2^ value for the test set greater than 0.6 were acceptable^40^. When 10% or 20% features were used (Table 1), the determination coefficients (*q*^2^, 10-fold cross-validation) of the training set ranged from 0.631 to 0.769, with an average value 0.671. The determination coefficients for the test sets were also encouraging (*r*^2^ = 0.607~0.766 with an average value 0.664). Therefore, the BRS-3D based regression models were acceptable. *RMSE* is also an important parameter for the prediction ability measurement. Even a model with low *r*^2^ can be practically useful if the *RMSE* is low^41^. The *RMSE* of the BRS-3D models were all lower than 1, which is acceptable since the data were collected from different research groups. The performance of 2B-3 selectivity regression model was the best one (*q*^2^_*cv*_ = 0.769, *RMSE* = 0.830 for training set and *r*^2^ = 0.766, *RMSE* = 0.828 for test set) among the six models.

**Figure 4:**
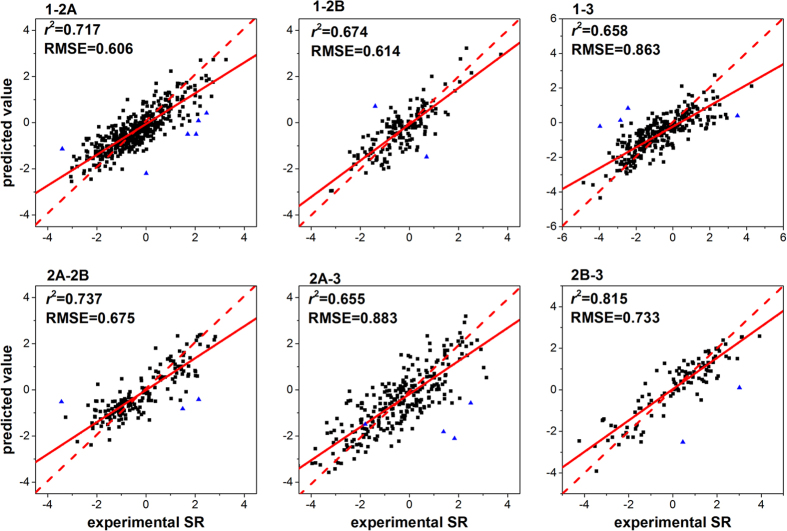
Correlation plots of experimental and predicted selectivity ratios of the test sets. The red dash straight line is the 45-degree benchmark line through the origin. The red solid straight line is fitting line of scatter diagram. Compounds outside the applicability domain were marked in blue.

The correlation plots showed good linear relationships between the experimental and predicted *SR* values (Fig. 4). The majority of the data points were concentrated around the 45-degree line through the origin, where the experimental and predicted *SR* values were equal to each other. The vertical distance from a symbol to the 45-degree line is the predicting deviation^41^. The fitting line indicated that the predicted *SR* values were close to the experimentally observed ones^21^.

### Model validations

Resampling strategy and Y-randomization test were used to assess the stability, validity and prediction ability of the models.

**Figure 5:**
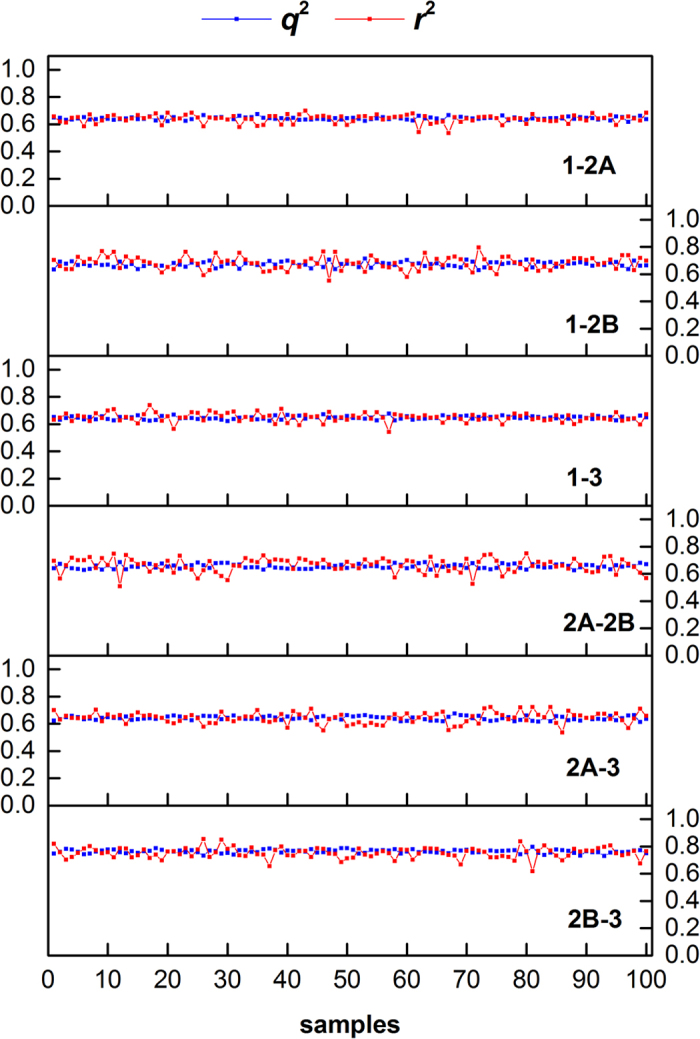
The 100 resampling models for subtype selectivity regression. The results showed that BRS-3D based models were stable.

First, resampling was applied to validate the stability of models. The data sets were randomly divided into training set and test set with the ratio of 4:1. The resampling were repeated for 100 times, which resulted in 100 models. The results of the resampling models were shown in Fig. 5. The prediction models were very stable both for the training sets and for the test sets. All the cross-validation *q*^2^ and *r*^2^ (test sets) were in the range of 0.6–0.8. Because *q*^2^ of the training sets were calculated with 10-fold validation, it was more robust than *r*^2^ of the test sets. The resampling results confirmed the robustness, stability and prediction ability of the BRS-3D based models.

**Figure 6:**
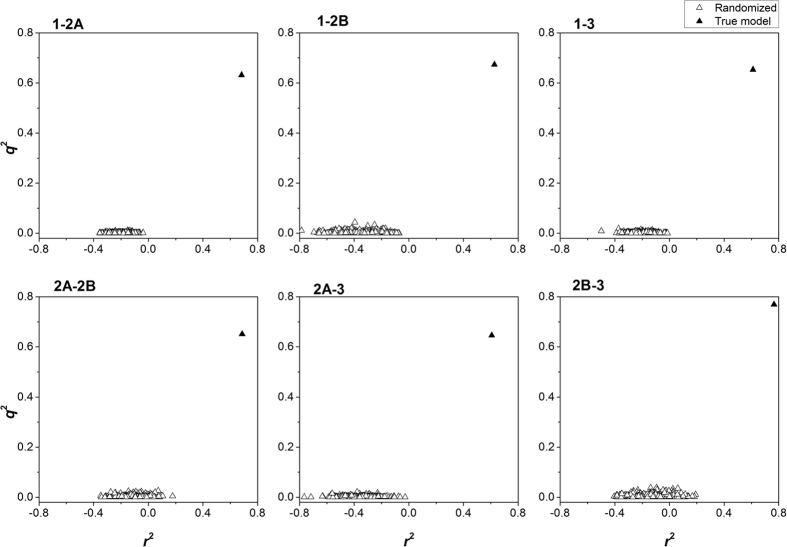
Y-randomization test of the selectivity regression models. The plot showed that the statistic results of true models (black triangles) were obviously better than the randomized models (hollow triangles).

Then, we conducted Y-randomization test (scramble stability test) to eliminate possible stochastic dependences. The distribution diagram of *q*^2^ and *r*^2^ values of the 500 randomized models and the true models were shown in Fig. 6. The *q*^2^ of randomly shuffled models ranged from 0 to 0.04, while the *r*^2^ ranged from −0.8 to 0.2. Hence, these models were totally without prediction ability. The statistically significant differences (Supplementary Table S2) between the shuffled models and the real models (*q*^2^ > 0.60, *r*^2^ > 0.60) confirmed the true association between the selected molecular descriptors and response property (*SR*) rather than chance correlation.

### Applicability domain evaluation

**Figure 7:**
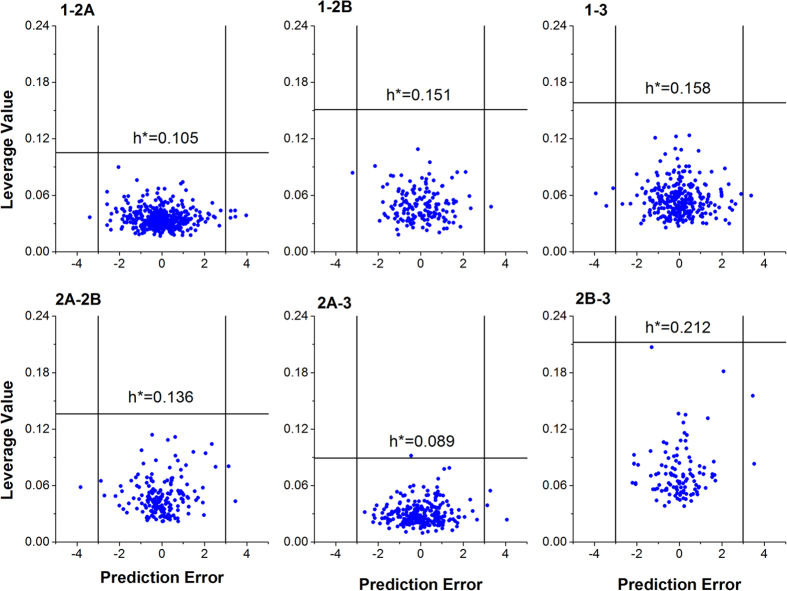
Williams plot of standardized residuals versus leverages for compounds in the test sets. The horizontal line shows the warning leverage (h* = 3*p/n*, *n* is the number of chemicals in training set and *p* is the number of variables plus one), the two vertical lines indicate the standardized residuals at 3 and -3 respectively. Most of compounds in the test sets fell within the AD of the models.

Williams plots were used to define the AD of the AR subtype selectivity prediction models (Fig. 7). The compounds outside the area formed by three black lines were identified as outliers. Most of the compounds in test sets fell within the AD. The test sets appear well distributed in the molecular descriptor space, it suggests that the predictive models developed with the training set can be applied to the test set.

### Comparison of BRS-3D and MOE 3D descriptors

BRS-3D is a shape similarity profile as molecular descriptor. We compared the prediction models built with BRS-3D and those built with the 3D molecular descriptors calculated with MOE program (Table 1). The results showed that the predictive ability of BRS-3D based models (average *q*^2^_*cv*_ = 0.671 and *r*^2^ = 0.664) performed better than or as good as MOE 3D descriptors (average *q*^2^_*cv*_ = 0.620 and *r*^2^ = 0.633).

### Pairwise subtype selectivity discrimination models

**Figure 8:**
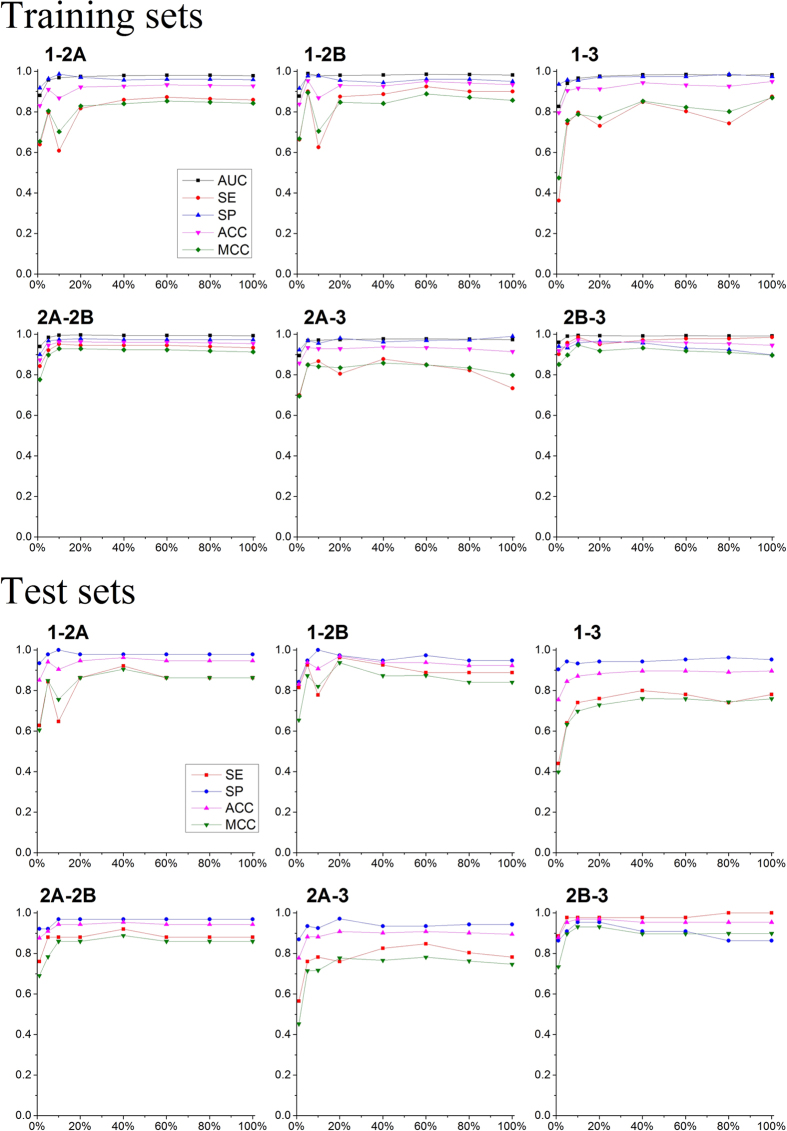
Feature selection of the six pairwise discrimination models. The parameters were calculated based on 10-fold cross-validation of the training set (top) or test set (bottom). The five symbols represent the area under the ROC (*AUC*), sensitivity (*SE*), specificity (*SP*), overall prediction accuracy (*ACC*) and Matthews correlation coefficient (*MCC*), respectively. Eight different feature subsets were explored. The test sets were used only for model evaluation.

We also developed six pairwise subtype selectivity discrimination models with 10-fold cross-validation and feature selection. The results of feature selection were shown in Fig. 8. As the results shown, with the increasing of BRS-3D features, the models showed a trend of increasing prediction accuracy. Using 5% or 10% features of BRS-3D can achieve acceptable prediction accuracy for most of the data sets. The fluctuation of the curves indicated that SVM was capable of dealing with high-dimensional data but was not robust to the presence of a large number of irrelevant descriptors. This situation explained the necessity of feature selection to multiple-dimensional molecular descriptor. Prediction results for the test sets with different feature subsets were also shown in Fig. 8. The results of the test sets showed similar trends with the training sets, which indicated the effectiveness of the cross-validation and there was no over-fitting in these models. The statistic results with 5% features were summarized in Table 2. For the training sets, the cross-validation *AUC* ranged from 0.940 to 0.991, indicating the high discriminate power of the models. The statistic results for the test sets, with *SE* = 0.640~0.977, *SP* = 0.909~0.978, *ACC* = 0.845~0.955 and *MCC* = 0.633~0.897 showed that the models’ prediction ability was acceptable. Among the models, 2B-3 pairs showed the best prediction results with *SE* = 0.977, *SP* = 0.909, *ACC* = 0.955 and *MCC* = 0.897 (test set).

Michielan *et al*. built a binary classifier for A_2A_ and A_3_ antagonists discrimination^21^. They used 3D auto-correlated electrostatic potential descriptors (autoMEP). The model was developed with SVM and LOO cross-validation. For training set (104 compounds), the over-all prediction accuracy (*ACC*_*cv*_) was 0.917. For test set (51 compounds), they reached a prediction of *SE* = 0.719, *SP* = 0.895, *ACC* = 0.784^21^,^22^. Our model (*ACC*_*cv*_ = 0.935, *SE*_*test*_ = 0.761, *SP*_*test*_ = 0.935 and *ACC*_*test*_ = 0.882) outperformed theirs, even we used a more diverse dataset (activity data in ChEMBL were collected from different research groups).

The results of discriminant models were consistent with the results of regression models. However, compared with the discrimination models, more compounds and activity information were used in the regression models. Therefore, we believe that the regression models were more predictive and practical, which can be confirmed with the high R^2^ and acceptable RMSE values. The discriminant models were provided to confirm the results of regression models.

### Model interpretation

**Figure 9:**
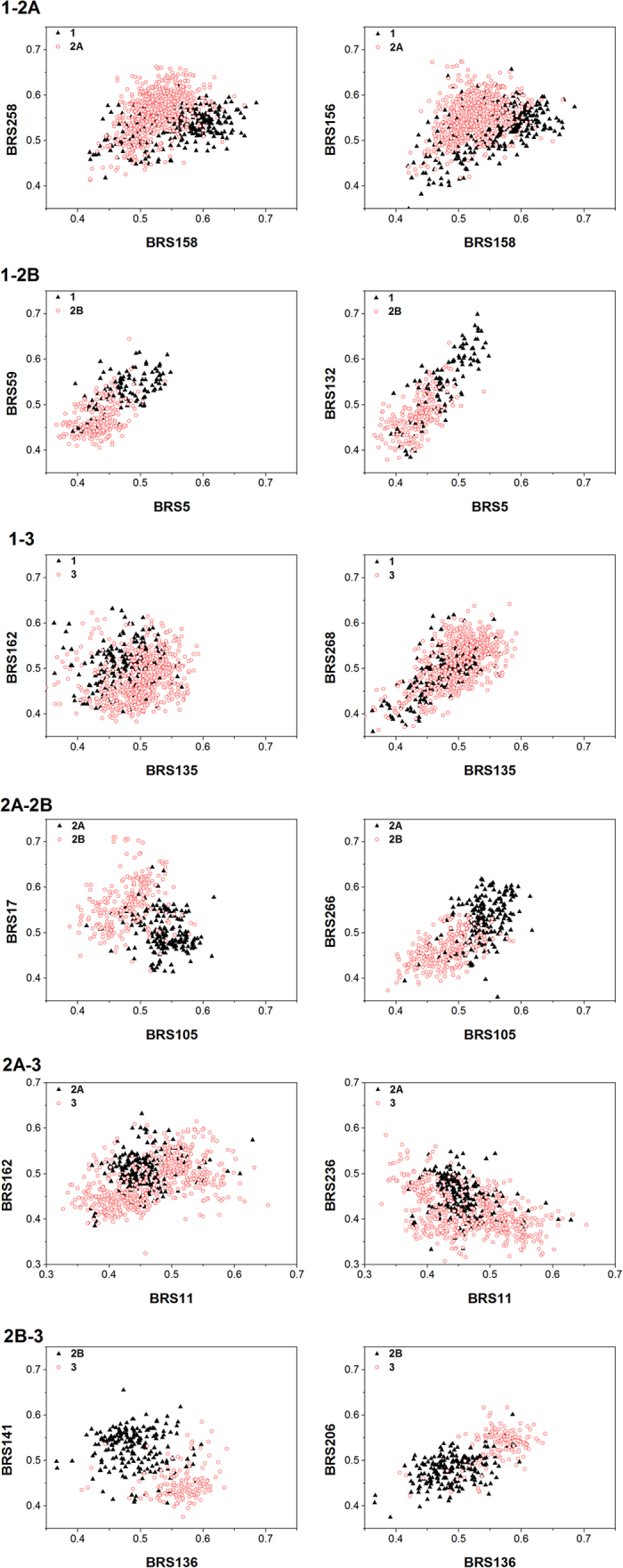
Distribution of the selective compounds in the shape similarity chemical spaces. The coordinates were defined as the most important BRS-3D features.

SVM based models can hardly be interpreted. Instead, we analyzed the distribution of the compounds in the chemical space composed with the most important features. As shown in Fig. 9, selective compounds again different targets distributed in different regions. For example, both the regression model and the discriminant model of the 2B-3 subtype pair showed good statistical results and prediction ability. Compounds similar to BRS141 (ligand IN7 from the *Homo sapiens* protease, PDB ID:1b8y) are more likely to bind with A_2B_, while compounds similar to BRS136 (ligand CTZ from the *Obelia longissima* Calcium-binding protein, PDB ID:1el4) and BRS206 (ligand OTT_PHE_SER_PRO_ALA_MAA_MP8 from the *Bacillus subtilis* protease, PDB ID:3kti) tend to bind with A_3_. The selective compounds cannot be distinguished with simple 2D or 3D properties (Supplementary Figure S2).

The information of the most important features was listed in Supplementary Table S3, and their corresponding ligands were listed in Supplementary Table S4. All the targets corresponding to the important features are irrelevant to AR, and there is no AR structures in the 300 BRCD-3D structures. Above results indicated that BRS-3D could be used for protein pocket similarity detection, e.g., the pocket of A_2B_ should be very similar to the pocket of *Homo sapiens* protease (BRS141, PDB ID:1b8y). We analyzed the superimposing conformations of the three most selective compounds in 2B-3 subtype pairs with the corresponding BRCD-3D ligands of the most important features (Supplenmentary Figure S3). The topological structures of the selective compounds are dissimilar to the BRCD-3D ligands. However, their 3D shapes are similar to each other according to the superimposition results. The results demonstrated the advantages of 3D methods than 2D ones.

**Figure 10:**
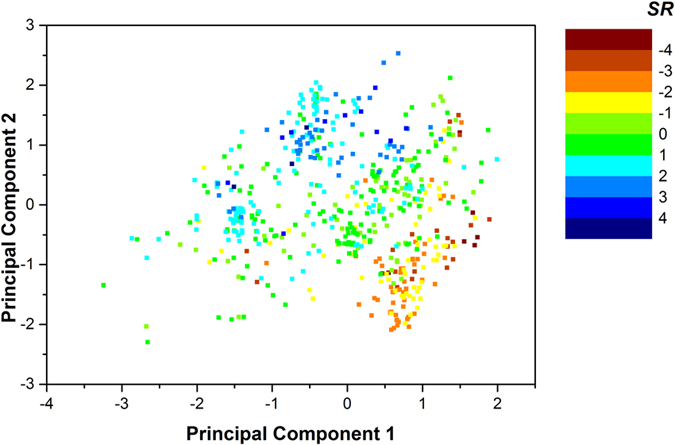
Distribution of the 2B-3 compounds in the space of the first two principal components. The compounds (dots) were colored according to their 2B-3 selective ratio (*SR*). The PCA analysis was carried out based on the 30 most important BRS-3D features in 2B-3 selectivity regression modeling.

We further performed a principal component analysis (PCA) over the 30 most important features that contributed to the 2B-3 regression model. The distribution of 2B-3 selective compounds in the coordinate plane of the first two principal components (variance explained: PC1 = 41.43% and PC2 = 16.82%) were shown in Fig. 10. We colored the dots (compounds) according to their experimental *SR*. The A_2B_ selective compounds (up-left) and the A_3_ selective compounds (bottom-right) were well separated with these two components.

It was assumed that the conformational transformation pattern plays an important role in subtype selectivity, while such pattern can be reflected with the BRS-3D. However, it should be noticed that not all the dots (compounds) in Fig. 9 were well distinguished. In fact, the selectivity is determined with lots of factors, for example, the pharmacophore distribution in 3D space. In such kind of situations, more BRS-3D components were needed to construct a predictable model, as the feature selection study indicated (Figs 3 and 8).

## Discussion

Target selectivity was a crucial requirement for drugs to avoid side-effects. It was commonly measured by the ratio of off-target *K*_i_ to the original target *K*_i_^46^. Many groups attempted to predict the selectivity of bioactive compounds^19^,^46^,^47^. However, theoretically predicting the subtype selectivity was very difficult^38^,^48^.

The recognition between the drugs and receptors is a process of 3D shape and property complementation. Therefore, the selectivity is mainly determined by the spatial arrangement of the drug’s functional groups, e.g., H-bond donors or receptors, charged centers. Compounds with different scaffolds tend to possess selectivity among different receptors, especially the inter-family systems. These systems could be theoretically studied with pharmacophore modeling or similar fixed-conformation approaches.

Hu *et al*. studied top-ranked intra- and inter-family target cliffs that formed by the largest number of selective compounds^25^. Intra-family target cliffs were generally associated with more compounds than inter-family cliffs. The study indicated that current researches were focused on intra-family selectivity. The intra-family selectivity is more complex, because different subtypes in the receptor family can be activated by the same substrate. We assumed that the intra-family selectivity was mainly determined by dynamic conformational transformation patterns of the ligands. Sophisticated molecule dynamic study could be applied in searching for the selective ligands for the intra-family systems. However, as we stated in the introduction section, receptor-based methods were limited by the availability of the receptor structures, accuracy of homology modeled structures and scoring function precision.

In this work, we introduced a novel multi-dimensional molecular descriptor, namely BRS-3D, for subtype selectivity prediction. BRS-3D was calculated by superimposing the objective compound onto 300 template ligands. Because the templates were diversely extracted from sc-PDB, the similarities in BRS-3D reflect the active conformation space of the objective compounds. Therefore, the descriptor can be applied well to conformation-related property prediction. As the results showed, through encoding multiple conformation information into the 300 dimensional descriptor, high predictive AR subtype selectivity models were developed. Even we used diverse data sets from the public available database (ChEMBL), our results were more predictive or comparable to earlier studies^21^. The method and models reported in this paper are helpful for further design and discovery of novel subtype specific AR agents.

BRS-3D is inherently three-dimensional molecular descriptor. Compared with 2D descriptors, it was considered to be suitable for scaffold hopping. Compared with commonly used 3D QSAR methods, e.g., CoMFA^49^, our approach is alignment independent. The BRS-3D models belong to the second class of QSAR models, according to the perspective by Fujita and Winkler^50^. When predictive models are constructed and validated, BRS-3D based virtual screening can be performed without human supervision. There are also some disadvantages of BRS-3D approach. First, molecular superimposition is computational resource consuming. Second, using of similarity array as molecular descriptor makes the interpretation of the prediction models very difficult. The models cannot provide effective guidance for novel molecule design.

In summary, through multiple conformation encoding, BRS-3D can be used as an effective molecular descriptor for AR subtype selectivity prediction. This unique approach can be integrated into the virtual screening workflow with other 2D, physicochemical properties or pharmacophore approaches.

## Additional Information

**How to cite this article**: He, S.-B. *et al*. Predicting Subtype Selectivity for Adenosine Receptor Ligands with Three-Dimensional Biologically Relevant Spectrum (BRS-3D). *Sci. Rep.* **6**, 36595; doi: 10.1038/srep36595 (2016).

**Publisher’s note:** Springer Nature remains neutral with regard to jurisdictional claims in published maps and institutional affiliations.

## Supplementary Material

Supplementary Information

## Figures and Tables

**Table 1:** The pairwise selectivity regression models based on BRS-3D and MOE-3D.

Targets	Descriptor	*N*^a^	*Training setb*[Fn t1-fn2]			*Test set*			*Test set*^c^		
			*Ntraining*	*q^2^cv*	*RMSE_cv_*	*Ntest*	*r^2^*	*RMSEtest*	*r^2^*	*RMSEtest*	*Nd*[Fn t1-fn4]
1-2A	BRS-3D	60	1746	0.631	0.729	436	0.683	0.656	0.717	0.606	6
	MOE-3D	42		0.559	0.794		0.577	0.758	0.614	0.710	9
1-2B	BRS-3D	30	617	0.673	0.756	154	0.627	0.657	0.658	0.863	2
	MOE-3D	44		0.665	0.766		0.553	0.719	0.615	0.910	2
1-3	BRS-3D	60	1158	0.653	0.874	290	0.611	0.941	0.674	0.614	4
	MOE-3D	38		0.545	0.990		0.577	0.981	0.664	0.596	7
2A-2B	BRS-3D	30	683	0.651	0.765	171	0.687	0.753	0.737	0.675	3
	MOE-3D	41		0.615	0.803		0.701	0.736	0.770	0.622	1
2A-3	BRS-3D	30	1048	0.646	0.982	262	0.607	0.950	0.655	0.883	4
	MOE-3D	41		0.601	1.041		0.633	0.919	0.672	0.874	5
2B-3	BRS-3D	30	438	0.769	0.830	110	0.766	0.828	0.815	0.733	2
	MOE-3D	42		0.734	0.887		0.757	0.845	0.794	0.779	3

^a^Number of features were determined with feature selection.

^b^Results of the training set were calculated based on of 10-fold cross-validation.

^c^Prediction results with test set compounds inside the applicability domain.

^d^Number of applicability domain excluded compounds.

**Table 2:** The pairwise selectivity discrimination models based on BRS-3D.

Targets	Features (%)^a^	*Training set^b^*[Fn t2-fn2]										*Test set*								
		*N_training_c*[Fn t2-fn3]	*TP*	*TN*	*FP*	*FN*	*AUCcv*	*SE*	*SP*	*ACCcv*	*MCC*	*Ntest*	*TP*	*TN*	*FP*	*FN*	*SE*	*SP*	*ACC*	*MCC*
1-2A	5	750	187	496	19	48	0.957	0.796	0.963	0.911	0.804	188	43	134	3	8	0.843	0.978	0.942	0.849
1-2B	5	260	72	176	4	8	0.989	0.900	0.978	0.954	0.895	65	25	36	2	2	0.926	0.947	0.939	0.873
1-3	5	622	113	450	20	39	0.940	0.743	0.957	0.905	0.757	155	32	99	6	18	0.640	0.943	0.845	0.633
2A-2B	5	356	153	184	6	13	0.985	0.922	0.968	0.947	0.898	89	22	59	5	3	0.880	0.922	0.910	0.784
2A-3	5	611	153	418	13	27	0.969	0.850	0.970	0.935	0.849	153	35	100	7	11	0.761	0.935	0.882	0.715
2B-3	5	262	137	111	8	6	0.991	0.958	0.933	0.947	0.898	66	43	20	2	1	0.977	0.909	0.955	0.897

^a^Number of features were determined with feature selection.

^b^Results of the training set were calculated based on 10-fold cross-validation.

^c^Abbreviations. *N*_*training*_: the number of compounds in training sets. *N*_*test*_: the number of compounds in test sets. *TP*: true positives. *FP*: false positives. *TN*: true negatives. *FN*: false negatives. *AUC*: the area under the ROC. *SE*: sensitivity. *SP*: specificity. *ACC*: overall prediction accuracy. *MCC*: Matthews correlation coefficient. cv: cross-validation.
