# Publisher Correction: Predicting Subtype Selectivity for Adenosine Receptor Ligands with Three-Dimensional Biologically Relevant Spectrum (BRS-3D)

**DOI:** 10.1038/s41598-021-85024-9

**Published:** 2021-03-05

**Authors:** Song-Bing He, Ben Hu, Zheng-Kun Kuang, Dong Wang, De-Xin Kong

**Affiliations:** 1 State Key Laboratory of Agricultural Microbiology, Huazhong Agricultural University, Wuhan, 430070 China; 2 College of Life Science and Technology, Huazhong Agricultural University, Wuhan, 430070 China; 3 Agricultural Bioinformatics Key Laboratory of Hubei Province, College of Informatics, Huazhong Agricultural University, Wuhan, 430070 China

Correction to: *Scientific Reports* 10.1038/srep36595, published online 04 November 2016

This Article contains errors. A Supplementary Dataset file, which was included with the initial submission, was omitted from the original version of this Article. The omitted Supplementary Dataset S1 is linked to this correction notice.

## Supplementary Information

Supplementary Information.

