# Supplementary material for: Predicting Subtype Selectivity for Adenosine Receptor Ligands with Three-Dimensional Biologically Relevant Spectrum (BRS-3D)
Source: Sci Rep. 2016 Nov 4;6:36595. doi: 10.1038/srep36595 (PMC5095671; doi:10.1038/srep36595)
Supplement: Supplementary Information [file srep36595-s1.pdf]

Supplementary Information

Predicting Subtype Selectivity for Adenosine Receptor Ligands with Three-Dimensional Biologically Relevant Spectrum (BRS-3D)

Song-Bing He[1,2], *Ben Hu*[3], *Zheng-Kun Kuang*[3], *Dong Wang*[2], *De-Xin Kong*[1,3,*]

[1]State Key Laboratory of Agricultural Microbiology, Huazhong Agricultural University, Wuhan 430070, China

[2]College of Life Science and Technology, Huazhong Agricultural University, Wuhan 430070, China

[3]Agricultural Bioinformatics Key Laboratory of Hubei Province, College of Informatics, Huazhong Agricultural University, Wuhan 430070, China

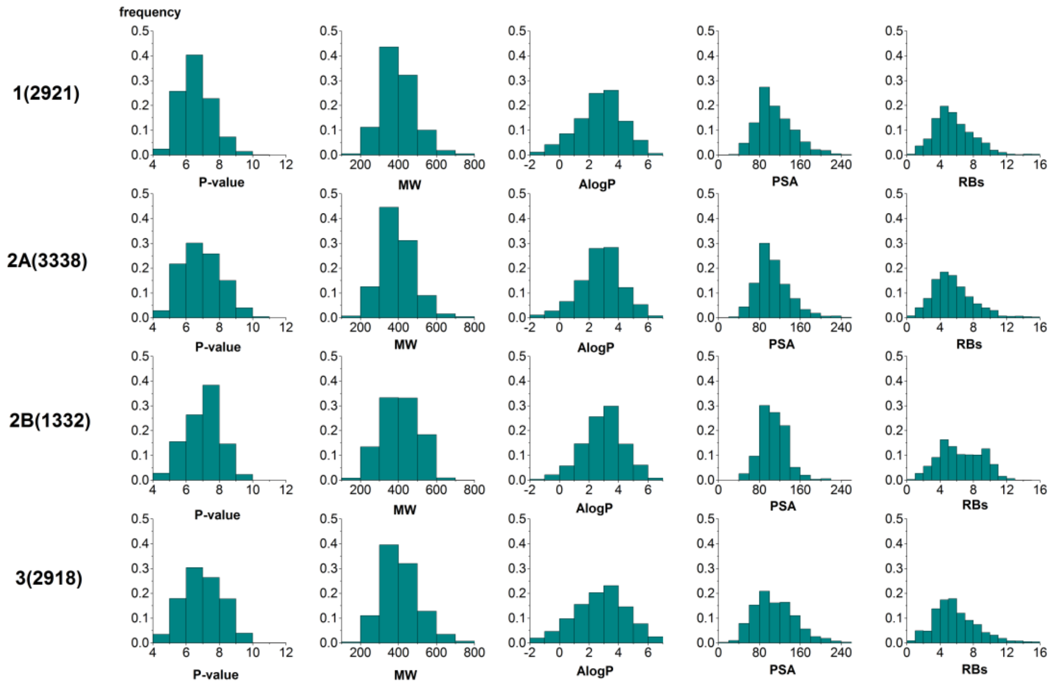

Figure S1. Activity and physicochemical properties (MW, AlogP, PSA, RBs) of the AR subtype active compounds. P-value: biological activity in pKi; MW: molecular weight; AlogP: the octanol/water partition coefficient; PSA: polar surface area; RBs: the number of rotatable bonds. All the physicochemical properties were calculated by Pipeline Pilot 8.5.

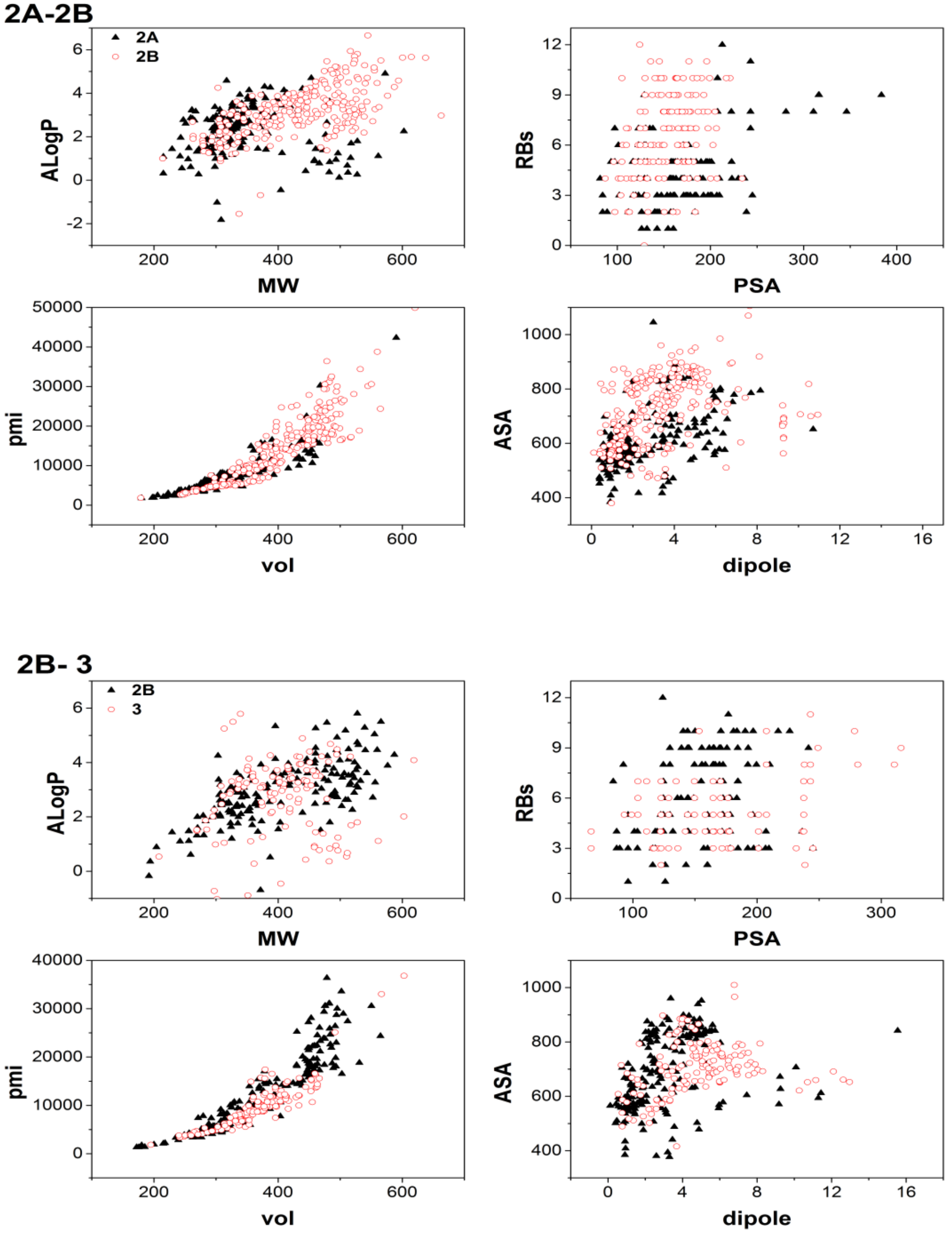

Figure S2. Distribution of the ligands in the physic-chemical property spaces for the 2A-2B and 2B-3 models. Activity and physicochemical properties: MW, AlogP, RBs, PSA; Typical MOE-3D molecular descriptors: vol, pmi, dipole, ASA. Each of these two subtype pairs can be distinguished with BRS-3D descriptors.

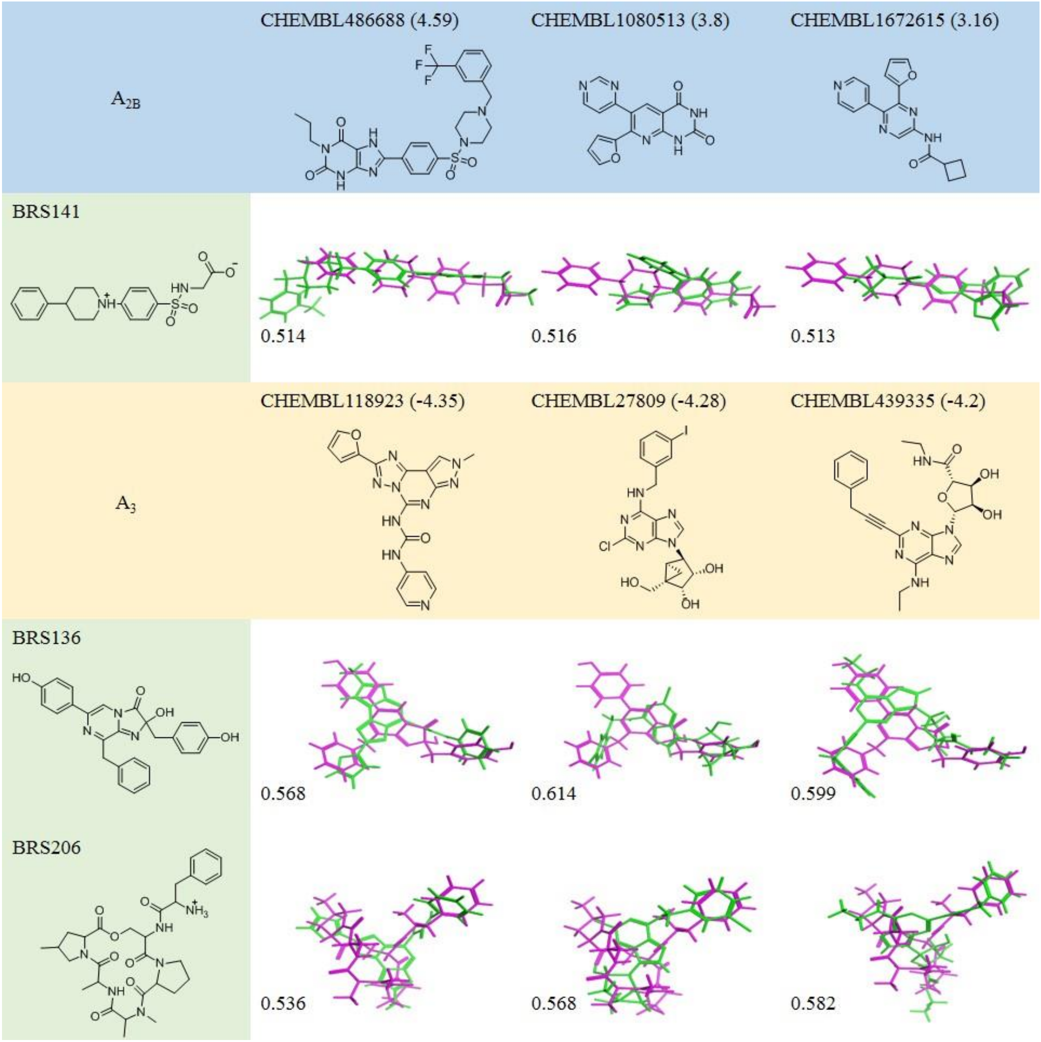

Figure S3. The superimposing conformations of the three most selective compounds of 2B-3 subtype pairs (green) with the corresponding BRCD-3D ligands of the most important features (purple). The values at the left bottom of alignment are the superimposition scores. The values in the bracket are the selectivity ratios of compounds.

Table S1. 3D molecular descriptors calculated by MOE.

Class	Types	N[a]	Descriptors
3D	Surface Area, Volume and Shape Descriptors	91	ASA,dens,glob,npr1,npr2,pmi,pmi1,pmi2,pmi3,rgyr,std_dim1,std_dim2,std_dim3,vol,VSA,vsurf_A,vsurf_CP,vsurf_CW1,vsurf_CW2,vsurf_CW3,vsurf_CW4,vsurf_CW5,vsurf_CW6,vsurf_CW7,vsurf_CW8,vsurf_D1,vsurf_D2,vsurf_D3,vsurf_D4,vsurf_D5,vsurf_D6,vsurf_D7,vsurf_D8,vsurf_DD12,vsurf_DD13,vsurf_DD23,vsurf_DW12,vsurf_DW13,vsurf_DW23,vsurf_EDmin1,vsurf_EDmin2,vsurf_EDmin3,vsurf_EWmin1,vsurf_EWmin2,vsurf_EWmin3,vsurf_G,vsurf_HB1,vsurf_HB2,vsurf_HB3,vsurf_HB4,vsurf_HB5,vsurf_HB6,vsurf_HB7,vsurf_HB8,vsurf_HL1,vsurf_HL2,vsurf_ID1,vsurf_ID2,vsurf_ID3,vsurf_ID4,vsurf_ID5,vsurf_ID6,vsurf_ID7,vsurf_ID8,vsurf_IW1,vsurf_IW2,vsurf_IW3,vsurf_IW4,vsurf_IW5,vsurf_IW6,vsurf_IW7,vsurf_IW8,vsurf_R,vsurf_S,vsurf_V,vsurf_W1,vsurf_W2,vsurf_W3,vsurf_W4,vsurf_W5,vsurf_W6,vsurf_W7,vsurf_W8,vsurf_Wp1,vsurf_Wp2,vsurf_Wp3,vsurf_Wp4,vsurf_Wp5,vsurf_Wp6,vsurf_Wp7,vsurf_Wp8

[a]Number of variables.

Table S2. Results of the Y-randomization test for selective regression models.

Target	Descriptor	Average q^2_{cv}	Average $RMSE_{cv}$	Average r^2	Average $RMSE$
1-2A	BRS-3D	0.001	1.306	-0.203	1.277
1-2B	BRS-3D	0.003	1.478	-0.353	1.250
1-3	BRS-3D	0.002	1.607	-0.202	1.653
2A-2B	BRS-3D	0.003	1.370	-0.095	1.407
2A-3	BRS-3D	0.002	1.870	-0.362	1.769
2B-3	BRS-3D	0.005	1.824	-0.125	1.814

$RMSE_{cv}$ and q^2_{cv} are root-mean-square error and squared correlation coefficients of 10-fold cross-validation for training sets, respectively. $RMSE$ and r^2 are root-mean-square error and determination coefficient for test sets, respectively.

Table S3. Three most important BRS-3D features for the six pairwise selectivity regression models.

Targets	BRS_ID	Ligand_Name	PDB_ID	Classification	Species
1-2A	BRS158	1z41_FMN	1z41	Oxidoreductases	Bacillus subtilis
	BRS258	2qu3_462	2qu3	Protease	Homo sapiens
	BRS156	2v7a_627	2v7a	Kinase	Homo sapiens
1-2B	BRS5	1b6h_LYS_NVA_LYS	1b6h	Peptide binding protein	Salmonella typhimurium
	BRS59	1ywr_LI9	1ywr	Kinase	Mus musculus
	BRS132	3i9o_AVW	3i9o	Hydrolases	Aplysia californica
1-3	BRS135	2fgi_PD1	2fgi	Kinase	Homo sapiens
	BRS162	3lc3_IYX	3lc3	Protease	Homo sapiens
	BRS268	2wzy_SQX	2wzy	Ligand-gated ion channel	Aplysia californica
2A-2B	BRS105	1r1h_BIR	1r1h	Protease	Homo sapiens
	BRS17	1j17_ZEN	1j17	Protease	Rattus norvegicus
	BRS266	2zog_BES	2zog	Protease	Mus musculus
2A-3	BRS11	1eno_NAD	1eno	Oxidoreductases	Brassica napus
	BRS162	3lc3_IYX	3lc3	Protease	Homo sapiens
	BRS236	2x2v_DPV	2x2v	Membrane protein	Bacillus pseudofirmus
2B-3	BRS136	1el4_CTZ	1el4	Calcium-binding protein	Obelia longissima
	BRS141	1b8y_IN7	1b8y	Protease	Homo sapiens
	BRS206	3kti_OTT_PHE_SER_PRO_ALA_MAA_MP8	3kti	Protease	Bacillus subtilis

Table S4. The ligand structures of three most important BRS-3D features for the six pairwise selectivity regression models.

1-2A	BRS158(1z41)	BRS258(2qu3)	BRS156(2v7a)
	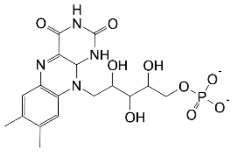	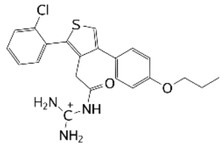	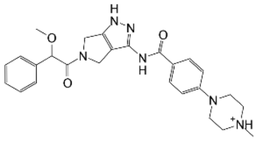
1-2B	BRS5(1b6h)	BRS59(1ywr)	BRS132(3i9o)
	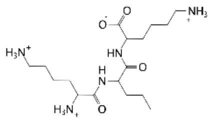	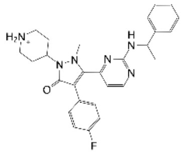	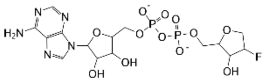
1-3	BRS135(2fgi)	BRS162(3lc3)	BRS268(2wzy)
	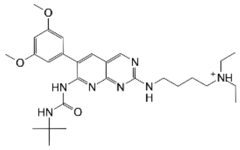	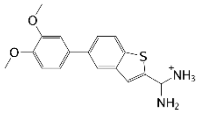	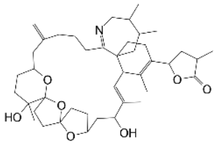
2A-2B	BRS105(1r1h)	BRS17(1j17)	BRS266(2zog)
	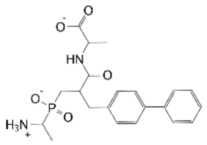	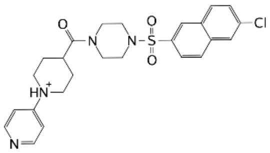	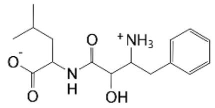
2A-3	BRS11(1eno)	BRS162(3lc3)	BRS236(2x2v)
	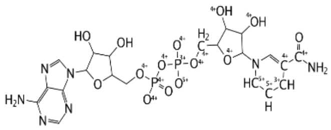	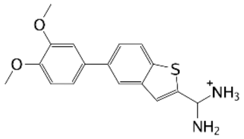	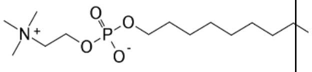
2B-3	BRS136(1el4)	BRS141(1b8y)	BRS206(3kti)
	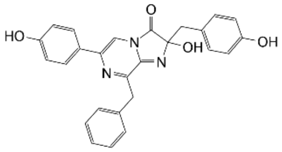	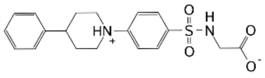	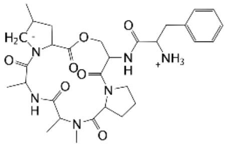

Supplementary Dataset S1

The dataset used in this study was provided separately as zipped SDF file.

The file contains all the information of adenosine receptor ligands, including ChEMID (Name), pKi values, selectivity ratio values and BRS-3D features.

The pKi values for each AR subtype are marked as pKi_A1, pKi_A2A, pKi_A2B and pKi_A3, respectively.
The SR values for each subtype pair are marked as SR_A1-A2A, SR_A1-A2B, SR_A1-A3, SR_A2A-A2B, SR_A2A-A3 and SR_A2B-A3, respectively.
BRS-3D scores of each compound are marked as BRS1, BRS2, BRS3 ... BRS300.
